# Mechanisms of Resistance to *Schistosoma japonicum* Infection in *Microtus fortis*, the Natural Non-permissive Host

**DOI:** 10.3389/fmicb.2020.02092

**Published:** 2020-09-03

**Authors:** Jia Shen, Suoyu Xiang, Mei Peng, Zhijun Zhou, Zhongdao Wu

**Affiliations:** ^1^Department of Parasitology, Zhongshan School of Medicine, Sun Yat-sen University, Guangzhou, China; ^2^Key Laboratory of Tropical Disease Control (SYSU), Ministry of Education, Guangzhou, China; ^3^Provincial Engineering Technology Research Center for Biological Vector Control, Guangzhou, China; ^4^Department of Laboratory Animal Science, Xiangya Medical College, Central South University, Changsha, China; ^5^Hunan Key Laboratory of Animal Models for Human Diseases, Changsha, China

**Keywords:** *Schistosoma japonicum*, resistance to infection, anti-infective factors, *M. fortis*, non-permissive host

## Abstract

Human schistosomiasis, which is caused by schistosomes, is a zoonosis that is difficult to control because of the many reservoir hosts. However, *Microtus fortis* is the only mammal that is naturally resistant to *Schistosoma japonicum* infection known in China, in which *S. japonicum* growth and development were arrested on day 12, and the worms eliminated on day 20 post-infection. In this review, we present an overview of the established and purported mechanisms of resistance to *S. japonicum* infection in *M. fortis* in comparison to *Rattus norvegicus*, a semi-permissive host. Clarifying the mechanism of this efficient resistance can help us to better understand host-parasite interaction and to provide better methods to control schistosomiasis.

## Introduction

Schistosomiasis, which is caused by schistosomes, is still one of the most serious zoonotic diseases, affecting over 200 million people in 78 countries. It is estimated that at least 229 million people required preventive treatment in 2018 (WHO, 2020). *Schistosoma japonicum* is one of eight schistosome species that infects humans. It is mainly prevalent in China, Indonesia, and the Philippines (WHO, 2020). In China, due to the implementation of a national program for disease control in the 1950s, *S. japonicum* infection is generally under control, and the reported number of cases was approximately 37,601 in China in 2017 (Li-Juan et al., 2018). However, it is difficult to eliminate this disease, because *S. japonicum* has many reservoir hosts. It has been reported that more than 46 species of mammals are susceptible to *S. japonicum* infection, such as cattle, goats and mice, in which worms will mature, lay eggs and form typical egg granulomas in the liver (He et al., 2001). However, some mammals show innate resistance to *Schistosoma* infection, such as *Rattus norvegicus* and *Microtus fortis* (*M. fortis*); the development of the parasites is prevented in these mammals, and the parasites cannot complete their lifecycle (Cioli et al., 1977; He et al., 1999b; He et al., 2001; Lu et al., 2011).

*M. fortis*, commonly known as the reed vole, is classified as Microtus, Cricetidae, Rodentia, Mammalia (ID: 100897) and distributed in China, Russia, North Korea and Mongolia (Batsaikhan and Tsytsulina, 2016). *M. fortis* is the only known mammalian host exhibiting strong natural resistance to *S. japonicum* infection (Li et al., 2000; He et al., 2001). This resistance can be inherited and is not affected by geographical distribution, environment (wild and laboratory-bred) or generation in *M. fortis* (Hongbin et al., 1995; Li et al., 2000). The migration of *S. japonicum* in *M. fortis* is similar to that in other hosts. A cercaria released from snails infects *M. fortis* via direct skin penetration, migrates through the lungs to the liver, and eventually dies in the liver (Li et al., 2000). When comparing the tegument of 10-days-old schistosomula collected from BALB/c mice or *M. fortis*, the schistosomula from *M. fortis* showed withering, sloughing, blebbing, and vacuole formation, and some structures (such as the muscle bundles and sensory organelles) were damaged (Peng et al., 2011). It has been reported that the growth and development of *S. japonicum* in *M. fortis* are arrested on day 12 and that worms subsequently atrophy and are completely disintegrated on day 20, dying 3–4 weeks after infection (Hongbin et al., 1995). Additionally, large numbers of bleeding points in the lungs of *M. fortis* have been found 6–10 days post-infection but are gone on day 15 post-infection (Li et al., 2000; Cheng et al., 2013). Interestingly, many white inflammatory nodules, composed of a schistosomulum surrounded by large numbers of inflammatory cells, have been observed on the surface of the liver in *M. fortis* at 12 days post-infection, and these nodules last until 42 days post-infection (Li et al., 2000; Cheng et al., 2013). The rapid elimination of *S. japonicum* in *M. fortis* indicates a highly effective immune response against *S. japonicum* infection in this rodent. Therefore, it is necessary to clarify the mechanisms underlying resistance development in *M. fortis*, which would contribute to our understanding of this specific host-parasite interaction but also provide a basis for the identification of vaccine candidates and novel drugs against schistosomiasis.

In this review, the established and purported mechanisms of resistance to *S. japonicum* infection in *M. fortis* are summarized and compared with those of rats.

## The Serum Components of *M. fortis* Involved in Resistance to *S. japonicum* Infection

It has been reported that the serum of *M. fortis* exerts a significant killing effect on schistosomula (the larval stage of *Schistosoma*) *in vitro* compared with that of mice (Xinyue et al., 2001; Liu et al., 2002; Yangli et al., 2003). Jiang et al. reported that passive transfer of serum from *M. fortis* to mice could protect the recipient mice against *Schistosoma* infection (Jiang et al., 2004). Their results showed that the number of worms in the mice that received the transfer was significantly reduced, the growth and development of schistosomes were obviously stunted, and the size of egg granulomas was significantly decreased compared with the corresponding parameters of control mice (Jiang et al., 2004). Therefore, these findings indicate that the serum components of *M. fortis* may be the key factors involved in the mechanism of resistance to *S. japonicum* infection in *M. fortis*.

## Antibodies

Antibody-mediated humoral immunity seems to play an important role in host defense. He et al. (1999a) found that there were natural antibodies against *S. japonicum* in *M. fortis*, including the antibodies of anti-soluble schistosomulum antigen (SSA), anti-adult worm antigen (AWA), anti-soluble egg antigen (SEA) and anti-cercarial antigen (CA), whose positive rates were 94.6, 85.5, 58.1, and 14.3%, respectively, which were significantly higher than those in mice. It is suggested that the acquired immunity of the *M. fortis* will increase rapidly after infection, which may play a role in resistance to *S. japonicum* infection. Jiang et al. found that the level of IgG3 in the serum of *M. fortis* was significantly higher than that in the serum of mice (Jiang et al., 2008). The IgG3 antibody levels against SSA and AWA in *M. fortis* were significantly increased by 79.6 and 49.6% in the 4th week post-infection, while no significant increase in IgG3 antibody levels was observed in BALB/c mice (Fu et al., 2001; Jiang et al., 2008). Moreover, the results showed that the mortality of schistosomula caused by IgG3 in wild and laboratory-bred *M. fortis* was 5.88 and 2.35 times higher than that in mice *in vitro* (Jiang et al., 2008). Furthermore, the numbers of worms were greatly reduced in infected mice after injection of IgG3 from *M. fortis* (Jiang et al., 2008). IgG3 has potent effector functions, such as complement activation, antibody-mediated phagocytosis, and antibody-dependent cell-mediated cytotoxicity (ADCC) (Damelang et al., 2019). Thus, it is suggested that IgG3 can effectively eliminate the worms and mediate protective immunity in *M. fortis*, which may be through activating complement system and ADCC effect mediated by immune cells.

In addition, the level of the transcript of immunoglobulin J polypeptide (*Igj*), which encodes the J chain protein of IgM and IgA antibodies, in the liver of *M. fortis* at 2 weeks post-infection was found to be higher than that in the liver of C57BL/6 mice (Hu et al., 2017), which indicated increased production of IgM and IgA. However, whether the levels of IgM and IgA in the serum of *M. fortis* are higher than those in the serum of mice has not been reported. Therefore, the roles of IgM and IgA in resistance to *S. japonicum* infection in *M. fortis* have not been determined.

## Complements

Complement is a system of blood plasma proteins that plays important roles in regulating inflammation, autoimmunity, and host defense (Holers, 2014; Merle et al., 2015; Copenhaver et al., 2019). Its role in the clearance of pathogens involves attracting leukocytes to sites of inflammation, increasing phagocytosis by myeloid cells, lysing microbes, and inducing B and T cell activation (Merle et al., 2015). Complement 3 (C3) and C4 are the most abundant complement proteins in the serum and play important roles in various functions of the complement system (Copenhaver et al., 2019). It has been reported that the levels of C3 and C4 in the serum of *M. fortis* are significantly higher than those in the serum of mice before infection and are increased in both hosts after infection with *S. japonicum* (He et al., 1998; Zhang et al., 2001). In addition, the expression of *C1qa* and *C3a* in the lungs or liver of *M. fortis* on days 6–14 post-infection was found to be significantly upregulated compared with that in infected mice (Jiang et al., 2010; Hu et al., 2017). Moreover, when comparing the killing effect of serum with or without complement from *M. fortis* or mice on schistosomula *in vitro*, Liu et al. found that the mortality rates of schistosomula in the group treated with serum from *M. fortis* infected with *S. japonicum* were 37.92% ± 16.36% and 40.15% ± 15.81% after incubation of serum and schistosomula for 16∼24 h and 40∼44 h, respectively, and these rates were significantly higher than those of the group treated with heat-inactivated serum from infected *M. fortis*, which were 13.40% ± 4.53% and 12.29% ± 1.84%, respectively (Liu et al., 2002). In contrast, in the group treated with serum from mice infected with *S. japonicum*, the mortality rates of schistosomula were 7.54% ± 1.85% and 16.39% ± 2.60%, respectively; these rates were not significant different from those of the group treated with heat-inactivated serum from infected mice, which were 7.23% ± 1.83% and 12.23% ± 1.94%, respectively (Liu et al., 2002). This study suggests that the rapid and effective elimination of *Schistosoma* in *M. fortis* may be dependent on complement. It is reported that IgG3 binds with a highly affinity to C1q, which form complexes to activate the classical complement pathway (Damelang et al., 2019). Therefore, complement mediated immunity against *Schistosoma* infection in *M. fortis* may be through the classical complement pathway activated by IgG3 to produce the membrane attack complex to lyse target cells in a schistosomulum.

## Albumin

Serum albumin, the most abundant protein in the circulatory system, is involved in many important physiological functions, such as maintenance of a stable plasma colloid osmotic pressure and the transportation, distribution and metabolism of many endogenous and exogenous substances (Fanali et al., 2012; Merlot et al., 2014). Moreover, albumin may play an important role in innate immunity (Gleeson and Dickson, 2015; Wilde and Katsounas, 2019). When schistosomula were cultured with purified albumin from the serum of *M. fortis* or a pcDNA3.1 plasmid encoding *M. fortis* albumin for 96 h *in vitro*, Li et al. (2013) found that the reduction rates of the schistosomula were 46.2 and 38.7%, respectively, which were significantly higher than the rate of the negative control group. Furthermore, when purified albumin from *M. fortis* was injected into infected mice, the worm burden was reduced by 43.5%, and the eggs per gram in the liver was decreased by 48.1% at 6 weeks post-infection in comparison to the corresponding parameters in control animals (Li et al., 2013). These results demonstrate that albumin in the serum may be an important factor involved in resistance in *M. fortis*. Interestingly, Li et al. (2013) reported that *M. fortis* albumin was poorly digested by the digestive system of *S. japonicum*. Thus, it seems that the inability of *S. japonicum* to survive in *M. fortis* is attributed to the difficulty of digesting albumin for *S. japonicum* during development (Li et al., 2013). Why can mouse albumin be digested by *S. japonicum*, while *M. fortis* albumin cannot? What is the difference in albumin between *M. fortis* and mice? These are interesting questions worth exploring from the perspective of genetic inheritance.

## Cytokines

Cytokines, which are secreted by immune cells (such as monocytes, macrophages, T cells, B cells, and natural killer cells) and some non-immune cells (endothelial cells, epidermal cells, fibroblasts, etc.), are a kind of small-molecule protein with wide-ranging biological activities, such as regulating cell growth, differentiation and maturation; regulating the immune response; and participating in inflammatory reactions (Hofmann et al., 2002; Borish and Steinke, 2003). It has been reported that interleukin (IL)-4, IFN-γ, TNF, IL-13, and IL-1 are involved in anti-infection effects (Borish and Steinke, 2003). Many studies have shown that cytokines also participate in resistance to schistosome infection. He et al. (1998) found that the level of IL-4 in the serum of *M. fortis* was 4 times higher than that in the serum of normal mice and showed an upward trend in both hosts after infection with *S. japonicum*. The level of IL-4 in the serum of *M. fortis* reached its peak at 9 days post-infection, increasing by 150.88%, which was close to the time when the development of schistosomula in *M. fortis* began to stagnate (11 days post-infection) (He et al., 1998). The results suggest that IL-4 may regulate the anti-*Schistosoma* immunity in *M. fortis*. In addition, Hu et al. found that the levels of IL-1β, GM-CSF, M-CSF, MCP-1, VEGF, IL-2, IL-12, IL-3, IL-4, IL-5, IL-10, IL-17, and IFN-γ in the serum of *M. fortis* were significantly higher at 2–3 weeks post-infection than in that of infected C57BL/6 mice (Hu et al., 2017). Moreover, genes related to these cytokines, including hepatocyte growth factor (*Hgf*), *C3a*, allograft inflammatory factor-1 (*Aif1*), lipopolysaccharide-binding protein (*Lbp*), and signal transducer and activator of transcription 6 (*Stat6*, the downstream mediator of Th2 cytokine signaling), exhibited significantly upregulated expression in the liver of *M. fortis* compared with that of mice at 2 weeks post-infection (Hu et al., 2017). Thus, these upregulated cytokines and their related genes may be involved in the resistance to *S. japonicum* infection in *M. fortis*. Although their exact roles need to be further elucidated, these results provide guidance for future research on this aspect of resistance.

## Immune Cells Participate in the Resistance to *S. japonicum* Infection in *M. fortis*

Immune cells generally participate in the anti-infection process by activating antigen-specific cytotoxic T lymphocytes, releasing various cytokines in response to antigens, performing antibody-dependent cell-mediated cytotoxicity (ADCC), opsonizing pathogens, etc. (Culley, 2009; Ochoa et al., 2017). The effector cells of ADCC are classically known to be natural killer (NK) cells, macrophages, neutrophils and eosinophils, which lyse target cells via specific antibodies bound to cell membrane surface antigens (Ochoa et al., 2017).

## Eosinophils

Eosinophils kill bacteria and parasites and play important roles in immune reactions and allergic reactions (Wen and Rothenberg, 2016). Wang et al. (2002) reported that a large number of eosinophils were infiltrated around worms in the lungs and the liver of *M. fortis* after infection with *S. japonicum* and that more eosinophils were present in the liver than in the lungs. Scanning electron microscopy showed that the development of the worms surrounded by eosinophils was abnormal and stunted. However, no obvious eosinophilic infiltration was found around worms in the tissues of mice at the same infection time because of the slight inflammatory response around the schistosomes in the mice (Wang et al., 2002). Moreover, Liu et al. found that a large number of eosinophils adhered to the surface of worms when eosinophils from *M. fortis* were co-cultured with schistosomula for 3∼6 h, and this number was larger than that of eosinophils from mice cultured under the same conditions (Liu et al., 2001). However, the relationship between schistosomulum mortality and eosinophil adhesion has not been observed *in vitro* (Liu et al., 2001). However, eosinophils cannot directly kill the schistosomulum, but they may arrest the schistosomulum through natural adhesion and combining with antibodies through ADCC. Nevertheless, reported results suggest that eosinophils may be an important effector cell in ADCC during anti-*Schistosoma* immunity in *M. fortis*.

## Lymphocytes

Lymphocytes, a kind of immune cell with immune recognition function, play an important role in defense against pathogen invasion and monitor cell variation *in vivo*; lymphocytes mainly include T lymphocytes, B lymphocytes and natural killer cells (Camara et al., 2012). Lymphocytes may participate in anti-*Schistosoma* immunity in *M. fortis*. Xinyue et al. (2001). found that schistosomulum mortality caused by lymphocytes from wild or laboratory-bred *M. fortis* was significantly higher than that caused by mouse lymphocytes when schistosomula were incubated with lymphocytes *in vitro* for 16∼18 h (Xinyue et al., 2001). After adding serum from *M. fortis* to the culture system, schistosomulum mortality was significantly increased, indicating that the serum and lymphocytes of *M. fortis* show a synergistic killing effect on schistosomes (He et al., 1998; Xinyue et al., 2001). Furthermore, Hu et al. reported that immunodeficient mice (nude mice and RAG-1^–/–^ mice) that received a bone marrow transplant (BMT) from *M. fortis*, which increased total lymphocyte, CD3^+^ T cell, B220^+^ B cell and CD3^+^CD8^+^ T cell frequencies, exhibited inhibition of the development of *S. japonicum* and a significant reduction in parasite spawning but did not show effects on the worm burden compared with immunodeficient mice that received a BMT from C57BL/6 mice (Hu et al., 2014). These results suggest that lymphocytes from *M. fortis* may affect the growth and development of *S. japonicum*.

## Macrophages

Although the general function of macrophages is promoting non-specific immunity, the roles of macrophages in ADCC and opsonization cannot be ignored (Campagne et al., 2007; Gul and van Egmond, 2015). Liu et al. (2001) reported that a large number of macrophages adhered to a schistosomulum after incubation of the schistosomulum with macrophages for 3∼6 h and that the number of adherent *M. fortis* macrophages was significantly higher than that of adherent mouse macrophages. This indicates that macrophages in *M. fortis* naturally possess the ability to adhere to schistosomula. However, the researchers did not find that this adhesion was related to schistosomulum mortality (Liu et al., 2001). Therefore, similar to eosinophils, macrophages may be an important effector cell in ADCC involved in arresting schistosomula in *M. fortis* but cannot directly kill the parasites.

## Micrornas May Play an Important Role in Resistance to *S. japonicum* Infection in *M. fortis*

MicroRNAs (miRNAs), a class of endogenous small non-coding RNA with a length of approximately 18–21 bp, regulate gene expression at the post-transcriptional level by degrading target mRNAs (Guo et al., 2010). miRNAs are involved in many important biological processes, such as development, proliferation, differentiation, apoptosis, energy metabolism and immunity (Tufekci et al., 2014). With microarray analysis, Han et al. (2013a) found that 162 miRNAs were differentially expressed between infected *M. fortis* and mice, with 12 in the liver, 32 in the spleen and 34 in the lungs in *M. fortis*. The functions of these miRNAs were found to be mainly involved in nutrient metabolism, immune regulation, etc. (Han et al., 2013a). Among the miRNAs, those that participated in nutrient metabolism were miR-143, which regulates adipocyte differentiation (Esau et al., 2004), and miR-375, which regulates glucagon levels and gluconeogenesis (Poy et al., 2009). The high levels of miR-705 and miR-122 together with the low expression of miR-193 and miR-27a may downregulate fatty acid synthesis and lipid metabolism, which will affect the lipid intake of *S. japonicum* in *M. fortis* (Han et al., 2013a). In addition, given that the food of *S. japonicum* is host erythrocytes, a high level of miR-223, which suppresses erythrocyte differentiation, in the liver in combination with low expression of miR-451, which promotes erythrocyte differentiation, could inhibit the development of *S. japonicum* by affecting the quality and quantity of erythrocytes in *M. fortis* (Felli et al., 2009; Patrick et al., 2010; Han et al., 2013a).

Some differentially expressed miRNAs in the spleen of *M. fortis* are involved in the inflammatory response and immune regulation, such as miR-15a regulating lymphoid development (Klein et al., 2010), miR-107 regulating macrophage adhesion (Hennessy et al., 2011), miR-125a-5p regulating the inflammatory response and lipid uptake (Chen et al., 2009), miR-1224 regulating the secretion of TNF (Niu et al., 2011), miR-150 promoting the differentiation of stem cells into megakaryocytes and controlling the differentiation of B cells and T cells (Gangaraju and Lin, 2009), miR-200a regulating the immune response (Chen and Mang, 2017) and so on (Han et al., 2013a); this differential expression pattern may play an important role in the resistance to *S. japonicum* infection in *M. fortis*.

Therefore, miRNAs that promote the clearance of *S. japonicum* in *M. fortis* are related to repression of nutritional components required by the parasite and participation in the immune response of the host.

## Elevated Metabolite Levels in *M. fortis* May Be Related to Resistance to *S. japonicum* Infection

When analyzing differential metabolites between infected *M. fortis* and mice, Hu et al. showed that the levels of quasi-vitamins (e.g., carnitine, stearoylcarnitine, palmitoyl-L-carnitine, and linoleyl carnitine), amino acids (N-acetylleucine, valine and leucine), unsaturated fatty acids and unsaturated fatty amines were significantly higher in the liver of *M. fortis* than in that of C57BL/6 mice (Hu et al., 2017). It has been reported that carnitines can increase CD4^+^ and CD8^+^ T cell levels during infection (Listed, 1998; Schutsky et al., 2014), that the related branched-chain amino acids can enhance the phagocytic function of neutrophils and the NK activity of lymphocytes (Tajiri and Shimizu, 2013; Nakamura, 2014) and that unsaturated fatty acids affect the function of neutrophils (Rodrigues et al., 2016). It seems that these metabolic molecules found at elevated levels can directly or indirectly improve the functions of host immune cells. Thus, these differences in metabolites may be related to the resistance to *S. japonicum* in *M. fortis*. Unfortunately, no experimental results are available to directly indicate that these metabolites are involved in the resistance to *S. japonicum* infection in *M. fortis*.

## Decreased Hormone Levels Impede the Development of *S. japonicum* in *M. fortis*

Previous studies have shown that *Schistosoma* parasites promote their growth and development via exploitation of host hormones, growth factors, etc. (Escobedo et al., 2005; Zhou et al., 2009; Aguilar-Díaz et al., 2015). Jiang found that the levels of thyroid hormone receptor alpha (*Thra*), thyroid hormone responsive protein (*Thrsp*) and steroid 11-beta dehydrogenase 1 (Hsd11b1) in the lungs and insulin-like growth factor 1 (*Igf1*) in the liver of *M. fortis* were significantly downregulated at 10 days post infection with *S. japonicum* (Jiang et al., 2010). It has been demonstrated that hyperthyroid mice harbor larger worms that mature earlier and produce more eggs, whereas the development of the worms is stunted in hypothyroid mice (Wahab et al., 1971; Saule et al., 2002). In addition, Brigg et al. discovered that *S. mansoni* could transform steroids of the host into their metabolites and that the number of eggs laid by *Schistosoma* parasites might be reduced when steroid hormones were inhibited, which suggested that steroids support the development of *S. mansoni* (Briggs, 1972). Igf-1, a growth hormone called somatomedin C, plays important roles in protein anabolism and the linear growth-promoting effect of pituitary GH (Aguirre et al., 2016). Indeed, Hu et al. identified some insulin receptors and growth factors of *S. japonicum* with high sequence similarity to those of mammals (Hu et al., 2003; Zhou et al., 2009). In addition, Khayath et al. (2007) also found two distinct insulin receptor homologs in *S. mansoni*, which were shown to interact with human proinsulin. These results imply that schistosomes can exploit the hormone system and other signal regulation systems of the host to support and promote their growth and development.

Therefore, the downregulated hormone-related genes in the lungs and liver of *M. fortis* may affect the growth and development of *S. japonicum* by reducing host-derived signals for growth and development. However, similar to the other mechanisms mentioned above, the exact roles of these hormones need further assessment in experiments.

## The Effector Molecules Participated in Protective Immunity Against *S. japonicum* in *M. fortis*

Heat shock proteins (HSPs), as molecular chaperones, have been shown to be potent activators of the innate immune system and are implicated in autoimmune diseases, antigen presentation and antitumor immunity (Tsan and Gao, 2004). *M. fortis* heat shock protein 90a (*Mf-HSP90a*), the homolog of HSP90a, has been shown to be a novel anti-*Schistosoma* molecule *in vitro* and *in vivo* (Gong et al., 2010). Co-culture of schistosomula with Mf-HSP90a-containing medium for 96 h was shown to cause 27% schistosomula mortality, which was significantly higher than that of the negative control (Gong et al., 2010). Furthermore, 40.8% worm burden reduction and 57.9% egg reduction in the liver were observed when an *Mf-HSP90*α expression vector was injected into mice (Gong et al., 2010).

Karyopherin a2 (KPNA2), a member of the karyopherin alpha family containing specific nuclear localization signals, functions with importin β to transport proteins into and out of the nucleus (Chook and Blobel, 2001). Cheng et al. (2011) reported that *M. fortis* Karyopherin a2 (*Mf-KPNA2*) caused a 15.8% schistosomula killing rate *in vitro*, a 39.42% worm burden reduction and a 76.50% egg reduction in the liver at 42 day post-infection *in vivo*. The results suggest a potent effect of Mf-KPNA2 on resistance to *S. japonicum* infection.

In addition, Wang H. M. et al. (2016) and Fan-Sheng et al. (2017) reported that *M. fortis* E77.43 (*Mf-*E77.43), a 12 kD protein isolated from the cDNA library of *M. fortis*, showed protective effects against *S. japonicum* infection. The results displayed that the recombinant Adeno-associated virus AAV2-MfE77.43 induced 27.3% reduction in worm load and 26.2% reduction in egg deposition in the liver of mice (Wang H. M. et al., 2016), while the recombinant retroviral vectors of pRevTRE-E77.43 caused 31% worm reduction and 35% egg reduction, respectively (Fan-Sheng et al., 2017).

## Apoptosis Governs the Elimination of *S. japonicum* in *M. fortis*

Apoptosis is a tightly regulated process by which cells undergo an inducible non-necrotic cellular death process to maintain the balance of cell proliferation and tissue remodeling activity in organisms (Jin and El-Deiry, 2005). Peng et al. (2011) reported that the percentages of early apoptotic and late apoptotic cells, as well as the level of caspase activity, in schistosomula from *M. fortis* were all significantly higher than those in schistosomula from BALB/c mice. In addition, by microarray analyses, Jiang et al. found that the levels of some apoptosis-inducing genes were significantly increased in the lungs and liver of infected *M. fortis* at 10 days post-infection compared to those of uninfected controls, including programmed cell death 6 (*Pdcd6*), caspase 7 (*Casp7*), and tyrosine protein kinase 2 (*Tyk2*) (Jiang et al., 2010). These apoptosis-promoting molecules can recognize programmed apoptotic protein receptors on the surface of pathological and aging cells and induce apoptosis (Jiang et al., 2010). Such apoptotic proteins may have the ability to initiate programmed cell death in *S. japonicum* during *M. fortis* infection. Therefore, it seems that this host can not only quickly activate its immune defense function but also initiate apoptosis in *S. japonicum* to eliminate the parasite after infection.

## Comparison of the Mechanisms Underlying Anti-*Schistosoma* Immunity Between *M. fortis* and Rats

Rats (*Rattus norvegicus*) are a semi-permissive host of *Schistosoma*, in which schistosomes fail to complete their life cycle (Cioli et al., 1977). When rats are infected with *Schistosoma*, most of the worms are eliminated at 4–6 weeks after the primary infection, and the eggs produced by the surviving worm pairs are limited and invalid (Cioli et al., 1977; Khalife et al., 2000). Moreover, rats also develop resistance to reinfection after primary infection (Knopf et al., 1977).

There are some similar phenomena in the mechanism of anti-schistosome infection between rats and *M. fortis*. It has been reported that passive transfer of serum from normal rats to mice could reduce the worm burden and egg production in the liver of the recipient mice (Zhou et al., 2001). In addition, the serum from rats showed a significant killing effect on schistosomula *in vitro*, while the killing effects was significantly decreased when incubated with heat-inactivated serum from normal rat (Wang et al., 2005), which shared the similar phenomena with *M. fortis.*

A comparison of the mechanism of resistance to *Schistosoma* infection between rats and *M. fortis* is shown in Table 1. The results showed that the humoral immunity plays an important role in this resistance in both hosts, but the involved natural antibodies, effector antibodies and effector cells are different. *M. fortis* is dependent on IgG3, eosinophils, macrophages and lymphocytes, while rats require IgG2a, IgE, eosinophils, macrophages, and platelets (Capron et al., 1978, 1983, 1984; Khalife et al., 2000), and mast cells enhanced the cytotoxicity to kill schistosomula in rats (Capron et al., 1978). In addition, it seems that the roles of cytokines, complements, oxidative stress, thyroid hormones and apoptosis in anti-*Schistosoma* immunity should not be ignored. In the rat model of schistosomiasis, primary infection and secondary infection induce a Th2 cytokine response characterized by increased expression of IL-4, IL-5, and IL-13, which is driven by egg production in mice (Cetre et al., 1999; Khalife et al., 2000). The worm burden was significantly increased only treated with anti-IL-4 R or anti-IL-13 R antibodies in rats, but not anti-IFN-γ R or anti-IL-5 R antibodies, suggesting a role of IL-4 and IL-13 in resistance (Khalife et al., 2000). Moreover, with iNOS^–/–^ rats, we demonstrated that the inherent high expression levels of iNOS in wild-type rats play an important role in inhibiting the development of *S. japonicum* (Shen et al., 2017). Induction of apoptosis in *Schistosoma* is also a resistance mechanism in rats. Wang et al. reported that apoptosis cells and the expression of apoptosis-associated genes in 42-day-old worms from rats were significantly increased compared with the worms from mice (Wang T. et al., 2016). On the other hand, it seems that the thyroid hormones in rats and *M. fortis* lead to opposite results: downregulation of thyroid hormone-related genes inhibits the development of *Schistosoma* in *M. fortis* (Jiang et al., 2010), whereas removal of the thyroid/parathyroid glands in rats improved the worm development (Knopf and Soliman, 1980). The reason may be that thyroid hormones are not only as growth factors for schistosomes but also as regulators and effectors that modulate the immune response of the host against the pathogen. Therefore, the interactions between *Schistosoma* and hosts are complex.

**TABLE 1:** Comparison of the mechanisms of resistance to *Schistosoma* infection between *M. fortis* and rat.

**Category**	***M. fortis***				**Rat**			
	**Components**	**Effects**	**Parasite species**	**References**	**Components**	**Effects**	**Parasite species**	**References**
Effector cells	Eosinophils, macrophages, lymphocytes	• Mediate ADCC effect to kill schistosomes; • Inhibit the development of schistosomes	*S. japonicum*	He et al., 1998; Liu et al., 2001; Xinyue et al., 2001; Wang et al., 2002; Hu et al., 2014	Eosinophils, macrophages, platelets, mast cells	• Mediate and enhance ADCC effect to kill schistosomes	*S. mansoni*	Capron et al., 1978, 1983, 1984; Khalife et al., 2000
Natural antibodies	Anti-AWA-Ab, anti-SEA-Ab, anti-SSA-Ab anti-CA-Ab	Maintain humoral immunity at a high level	*S. japonicum*	He et al., 1999a	Anti-AWA-Ab (IgG, IgG1, IgG2a), anti-SEA-Ab (IgG, IgG1, IgG2a, IgG3), anti-SSA-Ab (IgG1)	Maintain humoral immunity at a high level	*S. japonicum*	Wang et al., 2004
Effector antibodies	IgG3	• Mediate ADCC effect to kill schistosomes; • Activate complements; • Induce antibody-mediated phagocytosis	*S. japonicum*	Fu et al., 2001; Jiang et al., 2008	IgG2a, IgE	Mediate ADCC effect to kill schistosomes	*S. mansoni*	Capron et al., 1978, 1983, 1984; Khalife et al., 2000
Complements	↑ C3, C4	Produce MAC to lyse target cells	*S. japonicum*	He et al., 1998; Zhang et al., 2001	Complements in serum from normal rats	Produce MAC to lyse target cells	*S. mansoni* and *S. japonicum*	Ramalho-Pinto et al., 1978; Wang et al., 2004
Cytokines	↑ IL-4, IL-1β, GM-CSF, M-CSF, MCP-1, VEGF, IL-2, IL-12, IL-3, IL-4, IL-5, IL-10, IL-17 and IFN-γ	• Regulate cell differentiation and maturation; • Regulate the immune response	*S. japonicum*	He et al., 1998; Hu et al., 2017	↑ IL-4, IL-13	Control the production of IgE and eosinophils	*S. mansoni*	Cetre et al., 1999; Khalife et al., 2000
Hormones	↓Thra, Thrsp, Hsd11b1 and Igf1	Reduce host-derived signals for growth and development	*S. japonicum*	Jiang et al., 2010	Pituitary and thyroid/parathyroid hormones	Promote parasite elimination and inhibit worm development	*S. mansoni*	Knopf and Soliman, 1980
MicroRNAs	↑ miR-705, miR-122, miR-223; ↓miR-193, miR-27a, miR-451, miR-143, miR-375, etc.	• Regulate the immune response; • Downregulate fatty acid synthesis and lipid metabolism; • Affect the quality and quantity of erythrocytes	*S. japonicum*	Han et al., 2013a	↑ miR-346, miR-223, miR-720, miR-27a; ↓miR-691, miR-483, miR-467a, miR-196c, etc.	• Regulate the immune response; • Suppress the differentiation of erythrocyte and adipocyte	*S. japonicum*	Han et al., 2013b
Effective molecules	Albumin, HSP90a, KPNA2, *E77.43*	• Cause a difficult digestion; • Reduce worm burden and egg production	*S. japonicum*	Gong et al., 2010; Cheng et al., 2011; Li et al., 2013; Wang H. M. et al., 2016; Fan-Sheng et al., 2017	iNOS	Block the development of *S. japonicum*	*S. japonicum*	Shen et al., 2017
Apoptosis	↑ *Pdcd6*, *Casp7*, *Tyk2*	Initiate programmed cell death in *S. japonicum*	*S. japonicum*	Jiang et al., 2010	Induction of apoptosis in adult worms	Initiate programmed cell death in *S. japonicum*	*S. japonicum*	Wang H. M. et al., 2016

*ADCC, antibody-dependent cell-mediated cytotoxicity; AWA, adult worm antigen; SEA, soluble egg antigen; CA, cercarial antigen; SSA, soluble schistosomulum antigen; C3, Complement 3; C4, Complement 4; MAC, membrane attack complex; GM-CSF, granulocyte macrophage colony-stimulating factor; M-CSF, macrophage colony-stimulating factor; MCP-1, monocyte chemoattractant protein-1; VEGF, vascular endothelial growth factor; Thra, thyroid hormone receptor alpha; Thrsp, thyroid hormone responsive protein; Hsd11b1, steroid 11-beta dehydrogenase 1; Igf1, insulin-like growth factor 1; HSP90a, heat shock protein 90a; KPNA2, karyopherin alpha 2; iNOS, inducible nitric oxide synthase; Pdcd6, programmed cell death 6; Casp7, caspase 7; Tyk2, tyrosine protein kinase 2.*

## Conclusion and Perspectives

**FIGURE 1:**
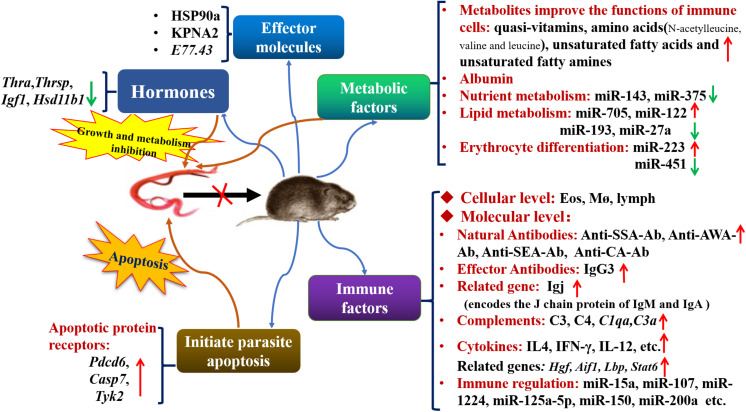
The established and purported mechanisms in anti-*Schistosoma* infection in *M. fortis.* Thra, thyroid hormone receptor alpha; Thrsp, thyroid hormone responsive protein; Igf1, insulin-like growth factor 1; Hsd11b1, steroid 11-beta dehydrogenase 1; Pdcd6, programmed cell death 6; Casp7, caspase 7; *Tyk2*, tyrosine protein kinase 2; Eos, eosinophil; Mø, macrophage; lymph, lymphocyte; SSA, soluble schistosomulum antigen; AWA, adult worm antigen; SEA, soluble egg antigen; CA, cercarial antigen; HSP90a, heat shock protein 90a; KPNA2, karyopherin alpha 2; Igj, immunoglobulin J polypeptide; C3, Complement 3; C4, Complement 4; C1qa, Complement 1q subcomponent subunit A; C3a, Complement 3 subcomponent subunit A; Hgf, hepatocyte growth factor; Aif-1, allograf inflammatory factor-1; Lbp, lipopolysaccharide-binding protein; Stat6, signal transducer and activator of transcription 6.

In summary, many factors in *M. fortis*, including an efficient immune defense system (e.g., antibodies, complements, cytokines, immune cells, MicroRNAs, pro-apoptotic mechanisms), high levels of metabolites, low hormone expression, and its albumin that is difficult for *S. japonicum* to digest, have developed an environment that is not suitable for the growth and development of *S. japonicum*. The established and purported mechanisms of resistance to *S. japonicum* infection in *M. fortis* are summarized and shown in Figure 1. And these results can provide guidance for future research in anti-infective immunity.

Although many mechanisms related to the resistance to *S. japonicum* infection in *M. fortis* have been proposed, further research is needed in this field, as some published results are limited to the observation of expression levels and lack sufficient supporting experimental data. The reason is that the research methods for *M. fortis* are mostly limited to microarray techniques and histological observation, and molecular biology methods are lacking owing to deficiencies in commercialized antibodies and transgenic *M. fortis* animals. The genomic information of *M. fortis* is still being analyzed. With the detailed genomic information, we can develop specific antibodies against *M. fortis* and use gene editing technologies such as CRISPR-Cas9 (Gupta et al., 2019) and/or RNAi to functionally analyze genes of interest and their role in resistance of *M. fortis* to *S. japonicum* infection. In addition, using CRISPR-Cas future research could also envisage to edit *S. japonicum* genes to study their relevance for host-parasite interaction in *M. fortis* versus other hosts.

In conclusion, it is necessary to further study the mechanisms of resistance in *M. fortis* and the *Schistosoma*-*M. fortis* interaction, which will deepen our general understanding of host-parasite interaction and host adaptability. Furthermore, the study on the mechanism of anti-schistosome infection and the *Schistosoma*-*M. fortis* interaction can provide important theoretical basis and new ideas for screening effective vaccine candidates and finding novel drugs against schistosomiasis by identifying effector molecules in the resistance.

## Author Contributions

JS and SX collected and reviewed the literature, and wrote the manuscript. MP collected and reviewed the literature. ZW and ZZ reviewed, edited, and approved its final version. All authors contributed to the article and approved the submitted version.

## Conflict of Interest

The authors declare that the research was conducted in the absence of any commercial or financial relationships that could be construed as a potential conflict of interest.
